# Coronary stenosis in Fabry disease with optical coherence tomography imaging presentation: a case report

**DOI:** 10.1093/ehjcr/ytag652

**Published:** 2026-09-08

**Authors:** Zesheng Peng, Bo Zheng, Yawen Zhao, Wei Zhang, Wei Ma

**Affiliations:** Department of Cardiology, Peking University First Hospital, No.8 Xishiku Street, Beijing 100034, China; Department of Cardiology, Peking University First Hospital, No.8 Xishiku Street, Beijing 100034, China; Department of Neurology, Peking University First Hospital, No.8 Xishiku Street, Beijing 100034, China; Department of Neurology, Peking University First Hospital, No.8 Xishiku Street, Beijing 100034, China; Department of Cardiology, Peking University First Hospital, No.8 Xishiku Street, Beijing 100034, China

**Keywords:** Case report, Fabry disease, Angina, Coronary stenosis, Optical coherence tomography

## Abstract

**Background:**

Fabry disease (FD) is a rare X-linked lysosomal storage disorder and also a rare cause of coronary stenosis. We present a case of coronary stenosis in a 39-year-old man with FD, including optical coherence tomography (OCT) imaging findings.

**Case summary:**

A 39-year-old man was admitted with unstable angina. He had been previously diagnosed with FD through α-galactosidase A enzyme activity testing and genetic analysis. Coronary angiography (CAG) revealed 90% stenosis in the proximal left anterior descending artery (pLAD). The pre-procedural CAG-based fractional flow reserve (caFFR) was 0.57, and the CAG-based index of microcirculatory resistance (caIMR) was 14.4. Drug-coated balloon angioplasty was performed, achieving post-procedural stenosis of 0%, with caFFR improving to 0.86 and caIMR decreasing to 9.4. He had no traditional atherosclerotic cardiovascular disease risk factors. Although CAG indicated 90% stenosis of the pLAD, OCT imaging did not demonstrate typical atherosclerotic or fibrocalcific plaques. However, based on the patient’s clinical features, OCT findings, and previous literature, we could not definitively determine the specific aetiology of the coronary stenosis (FD, coronary atherosclerosis, or both). During a 2-year follow-up, he remained angina-free.

**Discussion:**

Although OCT may aid in differentiating FD-related coronary involvement due to its high resolution and ability to detect FD-compatible changes, it cannot reliably determine whether the fibrous plaque morphology and loss of layered architecture are attributable to FD-related coronary pathology or premature atherosclerosis, representing a fundamental imaging limitation.

Learning points

- In patients with established Fabry disease presenting with coronary stenosis, the aetiology may be multifactorial, mainly including Fabry disease (FD)-related vasculopathy, accelerated atherosclerosis, or both, and should be evaluated accordingly.
- Optical coherence tomography can aid differential diagnosis of FD-related coronary lesions from atherosclerosis, but findings are non-specific and histopathology remains the gold standard.

## Introduction

Fabry disease (FD) is a rare X-linked lysosomal storage disorder caused by mutations in the *GLA* gene at Xq22.2. Deficiency of α-galactosidase A (α-Gal A) leads to pathological accumulation of globotriaosylceramide (Gb-3) and its deacylated metabolite, globotriaosylsphingosine (Lyso-Gb-3), in the cytosol and lysosomes, causing cellular damage and multi-system dysfunction.^1,2^ Although cardiac involvement is common and the leading cause of death in FD,^3^ FD is a rare cause of coronary stenosis. We present a case of coronary stenosis in a patient with FD, including optical coherence tomography (OCT) imaging findings.

## Summary figure

**Figure:**
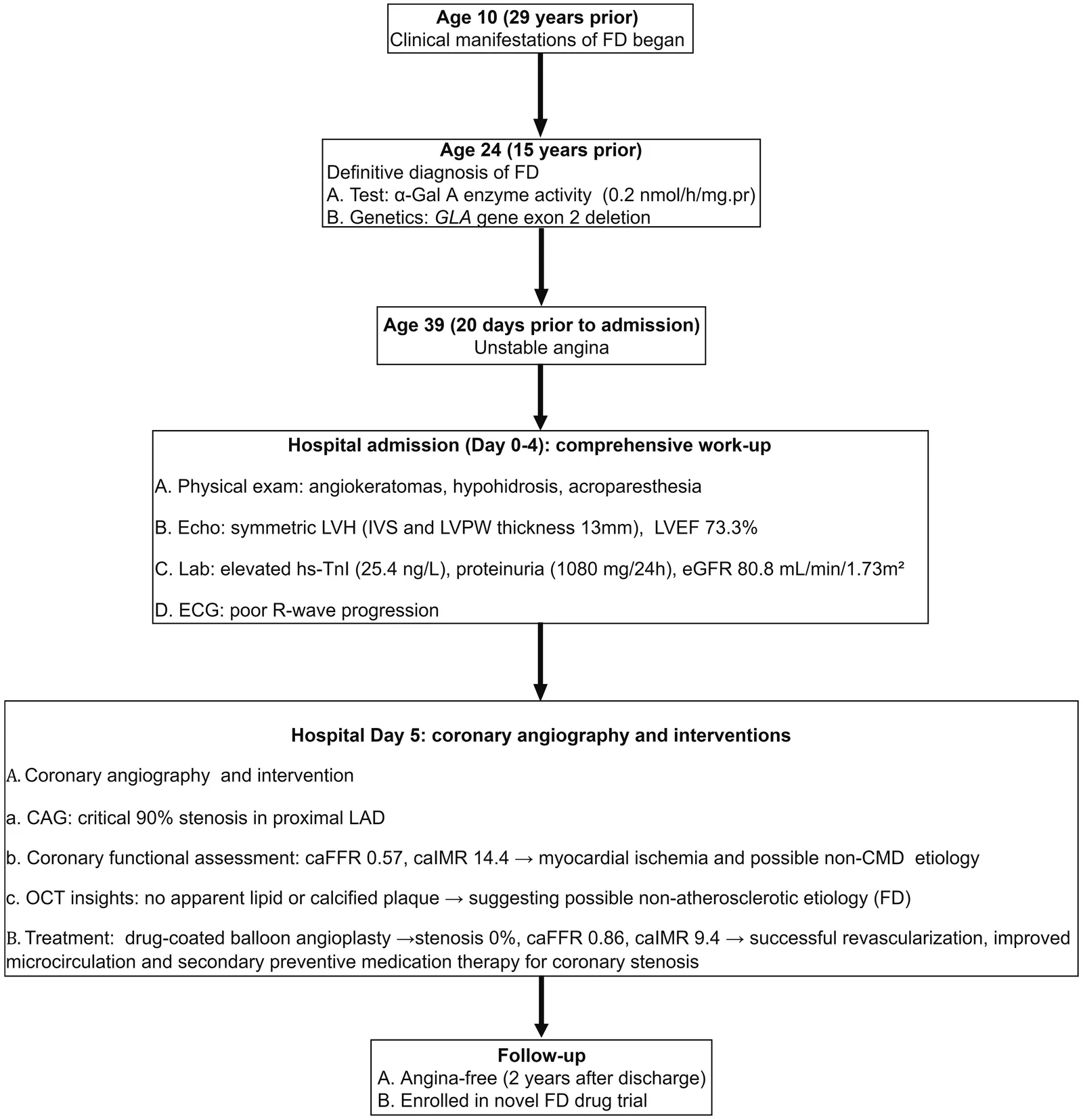


## Case presentation

A 39-year-old man presented with unstable angina 20 days prior to admission, manifesting as intermittent precordial pain during physical exertion. The pain was described as fist sized, occasionally radiating to the ulnar aspect of the left upper limb, and resolved after 3–5 min of rest. He first experienced FD-related symptoms 29 years ago, but the definitive diagnosis was not made until 15 years ago, based on α-Gal A enzyme activity [0.2 nmol/h/mg protein; normal range (NR) 29.0–64.4] and genetic analysis (*GLA* exon 2 deletion, a pathogenic mutation). Over time, he progressively developed FD-related multi-system involvement (*Table 1*) and denied any other significant medical history. He was married but had no children. Family history revealed that his mother and one maternal cousin had FD, and one maternal uncle with suspected FD had received both a pacemaker and a kidney transplant, ultimately dying from a cerebrovascular accident.

**Table 1:** Patient’s disease progression

Time point (years)	Clinical events and diagnostic findings
Nearly 10	Paroxysmal burning pain in palms and soles with hypohidrosis
24	Proteinuria, bilateral corneal epithelial radial opacities; FD diagnosed
28	Dizziness, vertigo, and nausea
29	Bilateral tinnitus (no hearing loss), worsening proteinuria; started ARB
34	Skin rash, left ventricular wall thickening, intracranial ICA small aneurysm, demyelinating neuropathy
36	Decreased right vertebral artery flow velocity; positive tilt table test confirming POTS
37	Acute vertigo, sudden sensorineural hearing loss, elevated cardiac troponin, increased bowel frequency
39	Coronary stenosis, sensorineural hearing loss, cerebral microbleeds and ischaemic foci, reduced LV strain on speckle-tracking echocardiography, eGFR 80.8 mL/min/1.73 m^2^

ARB, angiotensin receptor blocker; eGFR, estimated glomerular filtration rate; FD, Fabry disease; ICA, internal carotid artery; LV, left ventricular; POTS, postural tachycardia syndrome.

On admission, vital signs were as follows: temperature 37.3 °C, heart rate 110 b.p.m., respiratory rate 18 breaths/min, and blood pressure 122/75 mmHg. There were rice grain-sized erythematous papules at the left outer canthus, the left anterior tibial region, the abdomen, and the external genitalia (angiokeratomas); they were elevated and non-blanching. There were symmetrical flexion deformities of the distal interphalangeal joints of both little fingers. Lung sounds were clear, and heart sounds were normal without murmurs. The cardiac borders were not dilated, and there was no palpable hepatosplenomegaly. There was no lower extremity oedema. There was generalized xerosis and hypohidrosis of the extremities. No other significant abnormalities were noted.

Laboratory tests on admission showed the following: 24-h urine protein 1080 mg (NR 0–150), serum creatinine 100.51 μmol/L (NR 44–133), estimated glomerular filtration rate (eGFR) 80.8 mL/min/1.73 m^2^, total cholesterol 3.97 mmol/L, triglycerides 0.87 mmol/L, high-density lipoprotein cholesterol (HDL-C) 1.41 mmol/L, low-density lipoprotein cholesterol (LDL-C) 2.01 mmol/L, lipoprotein (a) (Lp(a)) not assayed, creatine kinase 87 U/L (NR 25–170), lactate dehydrogenase 192 U/L (NR 100–240), B-type natriuretic peptide (BNP) 43 ng/L (NR < 100), peak high-sensitivity troponin I 25.4 ng/L (NR 0–11.6), high-sensitivity C-reactive protein 2.93 mg/L (NR 0–3), and glycated hemoglobin A1c (HbA1c) 5.1% (NR 4%–6%). Other tests were unremarkable. The electrocardiogram (ECG) showed sinus rhythm with poor R-wave progression (*Figure 1*). Transthoracic echocardiography revealed symmetric left ventricular hypertrophy (LVH) (interventricular septum 13 mm, posterior wall 13 mm) and an left ventricular ejection fraction (LVEF) of 73.3%. Twenty-four-hour ambulatory blood pressure and ECG monitoring showed no significant abnormalities.

**Figure 1:**
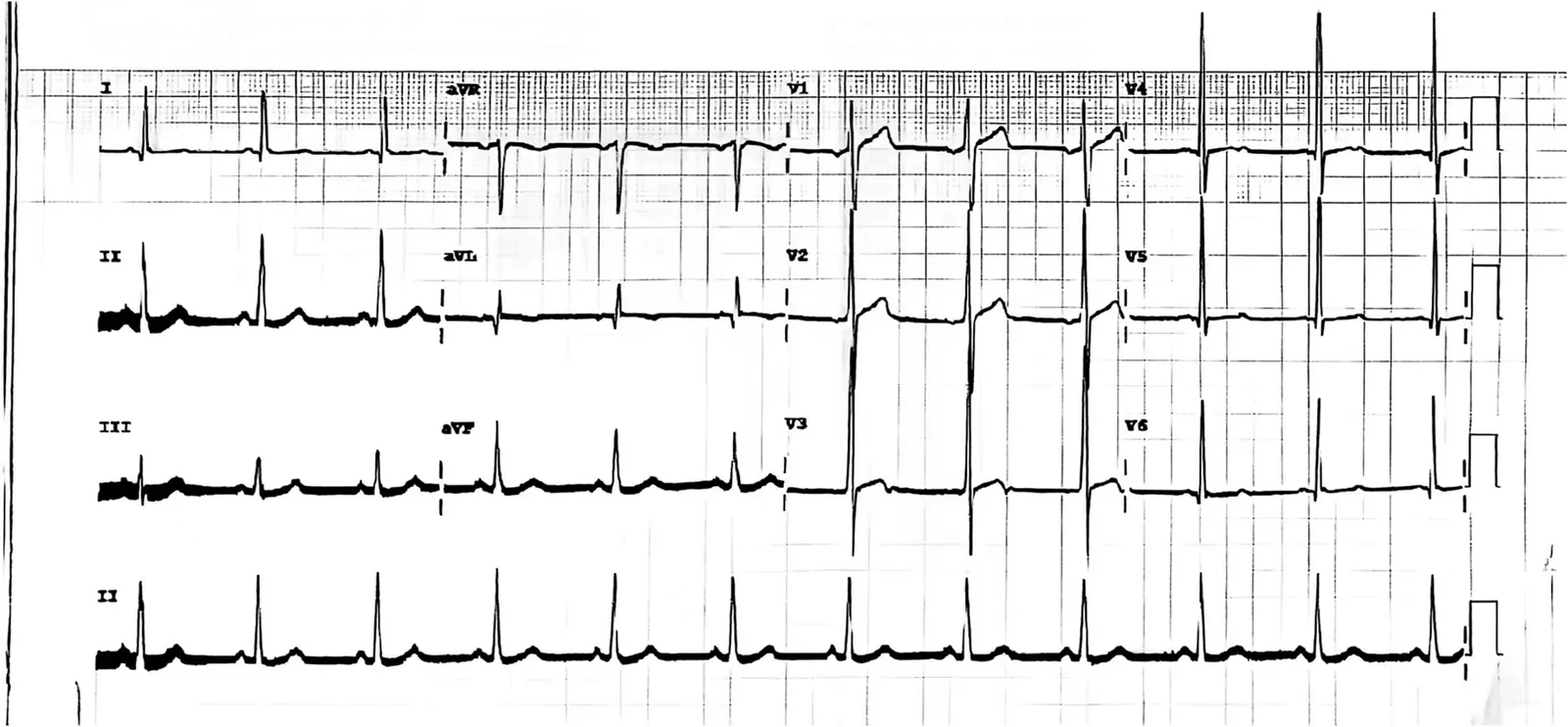
Electrocardiogram showing sinus rhythm with poor R-wave progression.

On hospital Day 5, coronary angiography (CAG) revealed 90% stenosis of the proximal left anterior descending artery (pLAD) and <50% stenosis in other coronary branches (*Figure 2*; Supplementary material online, *Video S1*). Pre-procedural CAG-based fractional flow reserve (caFFR) was 0.57 and CAG-based index of microcirculatory resistance (caIMR) was 14.4 (*Figure 3*). Although a drug-eluting stent would have been the preferred first-line treatment, the patient strongly declined a permanent stent. Consequently, drug-coated balloon (DCB) angioplasty was chosen after a thorough departmental discussion. Post-procedurally, CAG showed 0% stenosis of the pLAD (*Figure 2*; Supplementary material online, *Video S1*), and caFFR and caIMR were 0.86 and 9.4, respectively (*Figure 3*). The patient had no traditional atherosclerotic cardiovascular disease (ASCVD) risk factors (smoking, heavy alcohol, diabetes, dyslipidaemia, hypertension). Although CAG showed 90% pLAD stenosis, OCT did not reveal typical atherosclerotic or fibrocalcific plaques; however, it did demonstrate loss of the normal three-layered architecture, and the focal stenosis could be characterized as a fibrous plaque with fine, homogeneous texture (see Discussion). Based on the patient’s clinical features, OCT findings, and previous literature, he was diagnosed with coronary stenosis and FD with multi-system involvement, including the heart, kidneys, skin, autonomic nervous system, cerebrovascular system, eyes, and ears (*Table 1*). However, whether the coronary stenosis was attributable to FD, coronary atherosclerosis, or both remained unclear.

**Figure 2:**
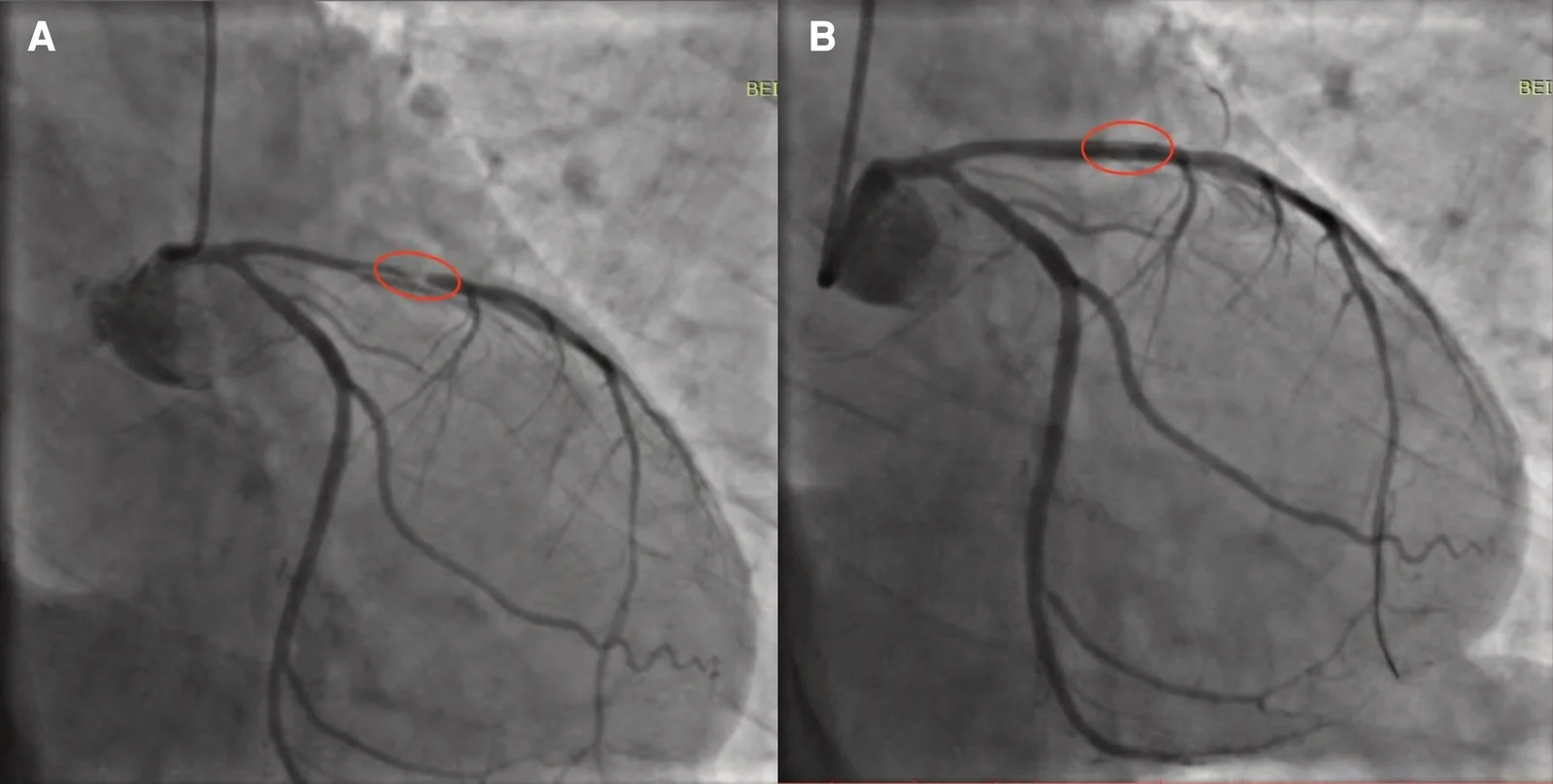
Coronary angiography showing 90% stenosis of the proximal left anterior descending artery before intervention (*A*) and 0% stenosis after drug-coated balloon angioplasty (*B*).

**Figure 3:**
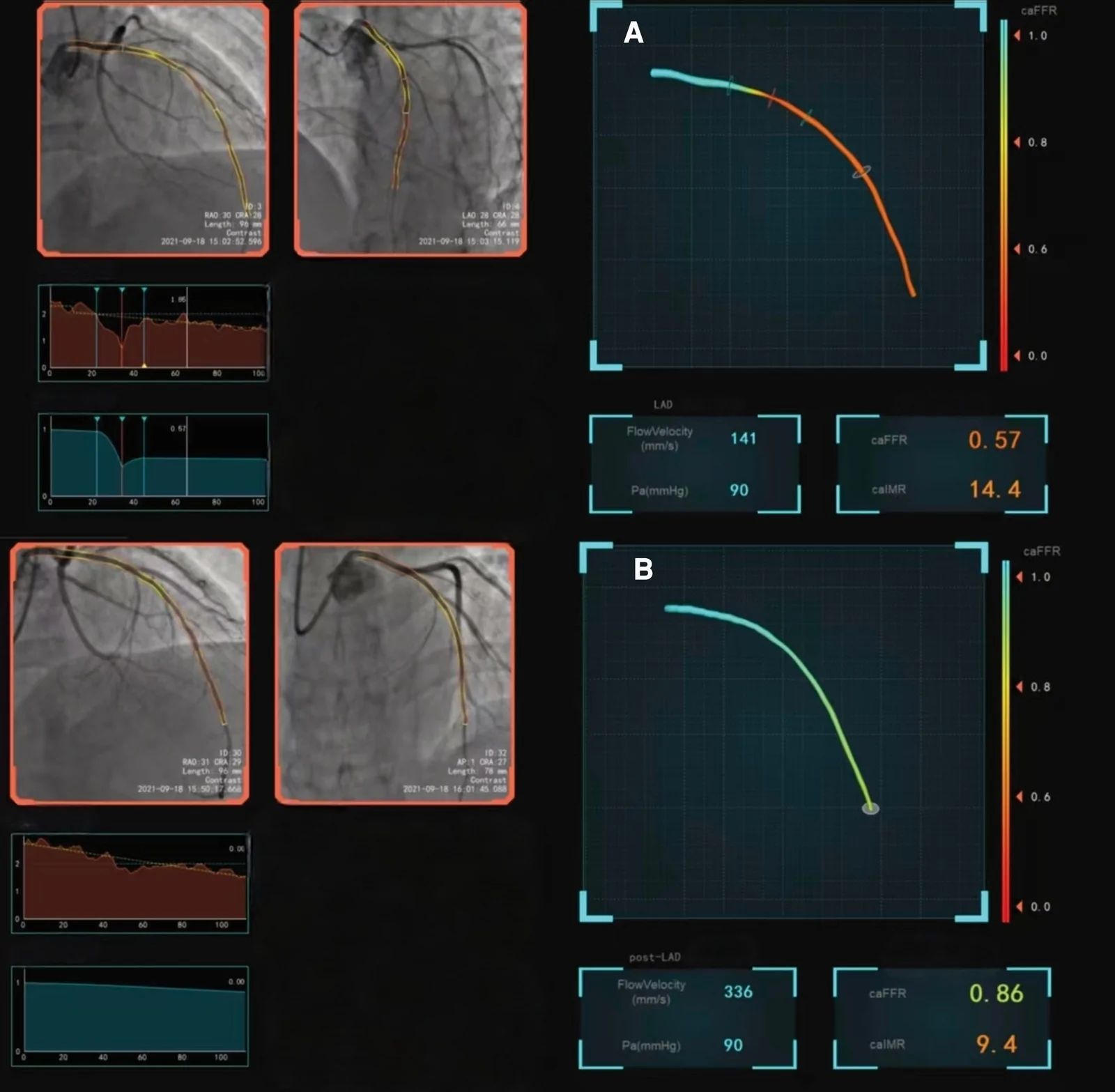
caFFR and caIMR. Pre-procedural caFFR and caIMR were 0.57 and 14.4 (*A*); post-procedurally, they were 0.86 and 9.4 (*B*). CaFFR = coronary angiography-based fractional flow reserve; caIMR = coronary angiography-based index of microcirculatory resistance.

Post-procedurally, he received standard secondary prevention (aspirin 100 mg once daily, clopidogrel 75 mg once daily, atorvastatin 20 mg at bedtime, losartan 50 mg once daily, rabeprazole 20 mg once daily). He later enrolled in a clinical trial of a novel FD drug (a new enzyme replacement therapy) and remained angina-free at the 2-year follow-up.

## Discussion

In FD, ∼22% of male patients (mean age 42 ± 5 years) and 23% of female patients (mean age 49 ± 13 years) may experience angina, which may arise from myocardial hypertrophy, coronary microvascular dysfunction (CMD),^4,5^ coronary spasm,^6^ or coronary stenosis.^7^ The present case presented with exertional angina, and no coronary spasm was seen on CAG, making spasm less likely.

Myocardial hypertrophy is the most common cardiac manifestation in ∼0.9% of patients with FD and often coexists with CMD when angina is present.^4,8^ In this case, echocardiography showed symmetric LVH. Although the pre- and post-procedural caIMR values were 14.4 and 9.4, respectively (threshold for CMD ≥ 25), caIMR is not equivalent to wire-based measurements, and a single caIMR measurement cannot definitively rule out CMD.

Previous literature has documented widespread glycolipid deposition in vascular smooth muscle and endothelial cells in FD.^9^ In coronary arteries, Gb-3 and Lyso-Gb-3 accumulation promotes hypertrophy and proliferation of vascular smooth muscle and endothelial cells, potentially increasing intima–media thickness and contributing to diffuse coronary stenosis^7,10,11^; however, the latter is rare. A cardiac pathology study^12^ found no occlusive atherosclerotic changes but did identify intimal thickening and/or medial hypertrophy in coronary vessels in FD. Autopsy reports indicate that coronary lesions in FD are often concentric with white discolouration, contrasting with the eccentric yellowish appearance of typical atherosclerotic plaques.^13^ Nevertheless, typical atherosclerotic plaques have been observed in FD,^7^ and it has been suggested that FD may accelerate atherosclerosis.^14^

We present OCT imaging of coronary stenosis in a patient with FD. Pre-procedurally, the minimal lumen area (MLA) at the focal stenosis was 1.01 mm^2^, with a fibrous cap thickness of 0.52 mm and a lipid arc of 360° (*Figure 4A*). No typical atherosclerotic or fibrocalcific plaques were seen; instead, OCT showed loss of the normal three-layered architecture, and the focal stenosis was a fibrous plaque with fine, homogeneous texture (see Supplementary material online, *Video S2*). Post-procedurally, MLA increased to 6.38 mm^2^ (*Figure 4B*), and the fibrous cap thickness at the proximal pLAD lesion was 0.45 mm (*Figure 4C*). Quantitative plaque composition could not be assessed due to equipment limitations.

**Figure 4:**
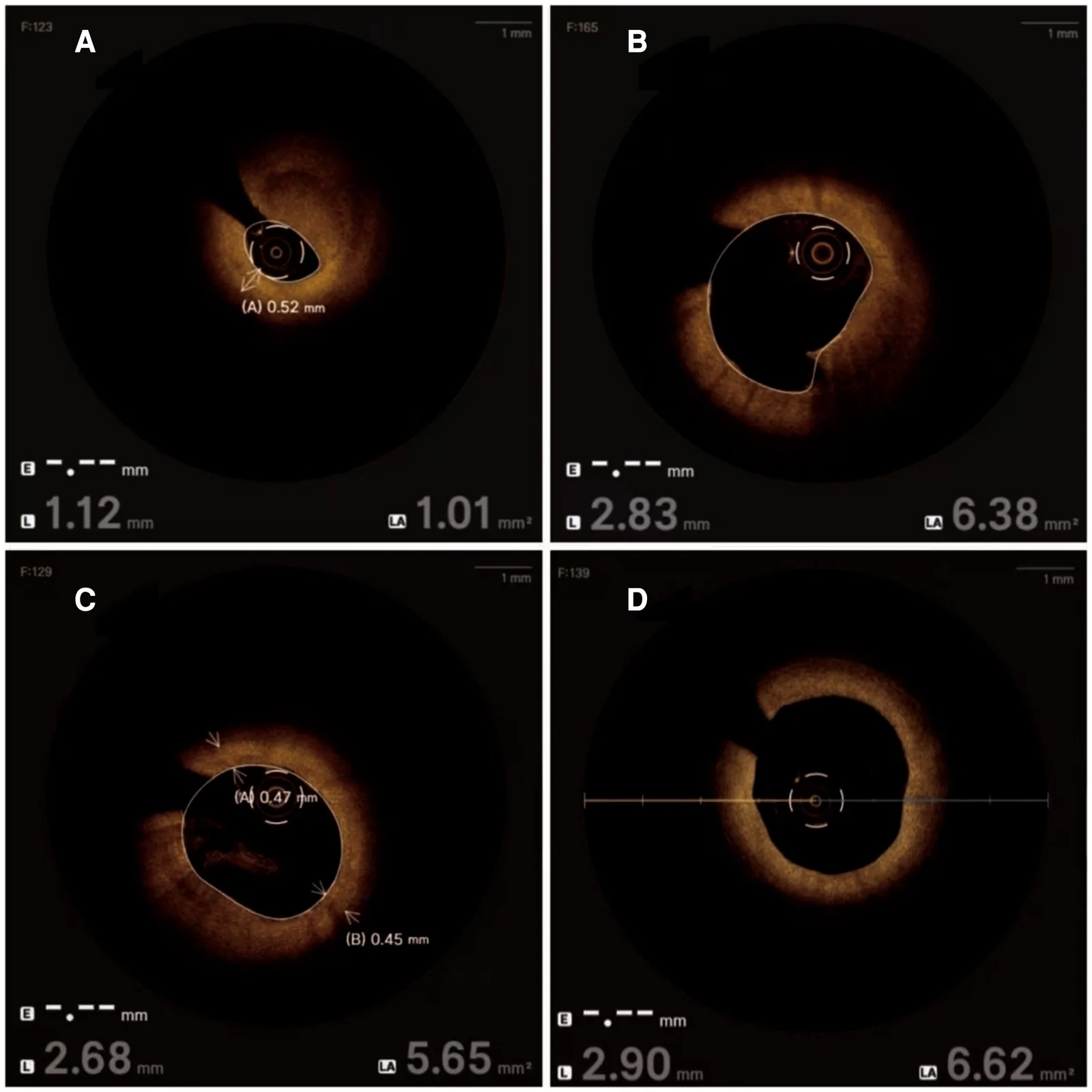
Optical coherence tomography (OCT) findings. Pre-procedural OCT of the focal stenosis in the proximal left anterior descending artery (pLAD) showing a minimal lumen area (MLA) of 1.01 mm^2^ and a fibrous cap thickness of 0.52 mm (*A*). Post-procedural OCT showing an MLA of 6.38 mm^2^ (*B*). Post-procedural OCT showing a fibrous cap thickness of 0.45 mm at the proximal pLAD lesion (*C*). Pre-procedural OCT of the distal segment of the focal stenosis revealing diffuse intimal thickening with deep signal attenuation and no distinct medial structure (*D*).

Overall, OCT revealed diffuse intimal thickening with deep signal attenuation and no distinct medial structure (*Figure 4A* and *D*), consistent with coronary artery pathology in FD (diffuse intimal and/or medial thickening with/without significant narrowing, thrombosis, or occlusion, often concentric).^12,13^ A focal stenosis was observed in the pLAD, with an eccentric high-signal plaque at the MLA site and a low-signal cavity from the 11 to 3 o’clock positions, suggestive of a healed plaque with possible organized thrombus—a finding inconsistent with typical atherosclerosis (*Figure 4B*). However, if the focal lesion truly represented a healed plaque, achieving 0% post-procedural stenosis would be unlikely.

While coronary stenosis is most often caused by ASCVD, rare causes, such as FD, should be considered in young patients presenting with angina, definite coronary stenosis, and a relevant family history but without traditional ASCVD risk factors. Although many clinical features of this case suggest a link between coronary stenosis and FD, the possibility of early or atypical atherosclerosis, or FD co-existing with early or atypical atherosclerosis, cannot be definitively excluded based on the available data (*Table 2*).

**Table 2:** Comparison of coronary artery changes among coronary atherosclerosis, Fabry disease, and the present case

	Coronary atherosclerosis	Fabry disease	The case
Typical atherosclerotic plaque	**+** ^15^	**+** ^7^	−
Intimal thickening	**+** ^15^	**+** ^7,10,11^	**+**
Concentric intimal thickening	**+** ^15^	**+** ^12,14^	**+**
Loss of layered architecture	**+** ^15^	**+** ^7,10,11^	+
Organised thrombus	**+** ^15^	−	±

‘+’ indicates presence; ‘−’ indicates absence or unknown.

Owing to its high tissue resolution and consistency with FD pathology as a whole,^12^ OCT is undoubtedly a valuable tool for differential diagnosis. However, the OCT pattern, specifically the fibrous plaque morphology and loss of layered architecture, cannot reliably distinguish FD-related coronary disease from premature atherosclerosis, representing a fundamental limitation of imaging-based diagnosis.

Regarding treatment, caFFR guided DCB decision-making for this pLAD lesion, as angiography alone is insufficient, and consensus recommends FFR ≥ 0.80 for DCB in large-territory lesions.^16^ For patients with unstable angina, potent P2Y12 inhibitors are preferred over clopidogrel unless contraindicated. This patient had no contraindication but had received aspirin and clopidogrel loading at the referring hospital. Post-procedural light transmission aggregometry showed adequate platelet inhibition (28%, therapeutic range 0%–50%). Clopidogrel was also more accessible locally. Rabeprazole was used for gastrointestinal protection due to fewer drug interactions.

We provide OCT imaging from a patient with FD for the first time, hoping to offer a new perspective for future clinical management. Further studies are needed to validate the OCT characteristics of FD-related coronary involvement.

## Supplementary Material

ytag652_Supplementary_Data

## Data Availability

The data and materials supporting this case report are not publicly available to protect patient privacy. De-identified data and materials are available from the corresponding author upon reasonable request.

## Lead author biography

Zesheng Peng is a resident physician in the Department of Cardiology at Peking University First Hospital. He is currently pursuing a master’s degree and undergoing standardized residency training at the same hospital.

## Notes

**Consent:** Written informed consent was obtained from the patient for publication of this case report and accompanying images, in accordance with COPE guidance.
